# Acute Refractory Hypocalcemia in a 51-Year-Old Male With a History of 1,1-Difluoroethane Inhalation

**DOI:** 10.7759/cureus.13693

**Published:** 2021-03-04

**Authors:** Evan Gleaves, Jacob M Nanney, Hassnain R Syed, Suneel Boyareddigari

**Affiliations:** 1 Internal Medicine, University of Kentucky, Bowling Green, USA; 2 College of Medicine, University of Kentucky, Lexington, USA

**Keywords:** drug abuse, internal medicine and endocrinology, difluoroethane, endocrinology

## Abstract

Hypocalcemia is a common electrolyte derangement that is most associated with parathryoid hormone or vitamin D abnormalities. Less common causes that most providers are aware of include hyperphosphatemia, acute pancreatitis, chronic kidney disease, and sepsis. However, certain populations are at risk for less common, but no less dangerous, causes. One such cause is 1,1-difluoroethane, an organofluorine that is used as a propellant in aerosol sprays and is commonly abused. 1,1-Difluoroethane has been noted to cause severe hypocalcemia by accumulation of the metabolite fluorocitrate in tissues. Here, we present the case of a 51-year-old male with severe hypocalcemia and multiple rib fractures following a fall, with recent history of tibial fracture. The patient had a medical history of osteoporosis with numerous fractures and chronic steroid use. He admitted to using keyboard cleaner as an inhalant for the previous month, which was found to contain 1,1-difluoroethane. Previous case reports on 1,1-difluoroethane inhalation have not reported a patient with preexisting osteoporosis or refractory hypocalcemia.

## Introduction

Difluoroethane is an organofluorine that is used as a refrigerant, foam expansion agent, and propellant for aerosol sprays. This class of chemical received widespread adoption as an alternative to chlorofluorocarbons. As such, it is found in many gas dusters and other consumer aerosol products. On inhalation, it causes acute onset of euphoric effects and rapidly enters the bloodstream [1]; therefore, it has become increasingly popular as a recreational drug. Adverse effects from difluoroethane inhalation vary based on acuity and dose, including nausea, vomiting, altered mental status, seizure, acute kidney injury [2], hypocalcemia, skeletal fluorosis [3], and cardiac arrhythmias [4,5]. Here, we present the case of a 51-year-old male who presented with adverse effects including severe hypocalcemia and multiple rib fractures following a fall, with a recent history of tibial fracture. The patient had a past medical history of osteoporosis with numerous fractures and chronic steroid use. He admitted to using keyboard cleaner as an inhalant for the previous month, which was found to contain 1,1-difluoroethane.

## Case presentation

Our patient was a 51-year-old male with a medical history of osteoporosis, chronic steroid injections, falls, polysubstance abuse, chronic back pain, and multiple fractures. He presented to an outside facility complaining of left-sided rib pain after a fall which the patient stated occurred while he was huffing keyboard cleaner. He had undergone treatment for a right leg fracture several weeks before and admitted to smoking marijuana and “huffing” for pain control. An X-ray revealed acute fractures of the fifth through eighth ribs on the left, along with old fractures of the third through sixth ribs of the left, and fifth through eighth on the right. He was transferred to our facility for possible rib plating. Initial laboratory values were significant for white blood cell count 9,500/uL (normal: 4,800-10,800/uL), hemoglobin 12.8 g/dL (normal: 13.0-18.0 g/dL), calcium 4.0 mg/dL (normal: 8.4-10.2 mg/dL), corrected calcium 3.9 mg/dL (normal: 8.3-9.7 mg/dL), vitamin D 25-hydroxy 26 ng/mL (normal: 30-100 ng/mL), phosphorus 2.6 mg/dL (normal: 2.5-4.5 mg/dL), and parathryoid hormone (PTH) 475 pg/mL (normal: 7.5-53.5 pg/mL). The patient displayed no symptoms of hypocalcemia. Over the course of nine days, he received 36 g of calcium gluconate IV, 2 g calcium chloride IV, and 5.6 g calcium carbonate PO, which succeeded in bringing him to corrected calcium of 8.0 mg/dL. His PTH reduced to 301.6 pg/mL upon recheck on day eighth of his admission. He remained asymptomatic aside from oral mucosal and rib pain during this time. The patient was discharged from our facility with plans to follow-up with endocrinology for further workup of his hypocalcemia and hyperparathyroidism. The patient was regrettably lost to follow-up.

## Discussion

Our patient had severe refractory hypocalcemia that is atypical for acute difluoroethane inhalation, possible exacerbating factors include vitamin D deficiency, longer-term inhalant abuse than admitted, and chronic spinal steroid injections, which were performed due to chronic back pain from a motor vehicle collision. Serum 25-hydroxyvitamin D levels greater than 20 ng/mL are generally considered adequate for bone and overall health in healthy individuals [6]. Vitamin D deficiency has been shown to induce hypocalcemic seizures with calcium levels as low as 6.4 mg/dL [7]. Our patient presented with a calcium of 3.9 mg/dL, which is below the value that would be explained by vitamin D deficiency alone. Our patient’s hyperparathyroidism is potentially explained by simple hypocalcemia, vitamin D deficiency, or skeletal fluorosis, which has previously been reported secondary to long-term difluoroethane abuse [1]. It is likely that his hyperparathyroidism was secondary to hypocalcemia as his PTH level was within the expected range that can occur from hypocalcemia, particularly in the acute setting [8]. It additionally responded to increasing serum calcium levels, as seen in the later PTH value. The mechanism of hypocalcemia from 1,1-difluoroethane is similar to that of other fluorinated ethanes. Once inhaled, fluoroethane is metabolized to fluoroacetate via an aldehyde or acyl fluoride, which complexes with coenzyme A and is metabolized by citrate synthase into fluorocitrate. Fluorocitrate can accumulate in multiple tissues and lead to hypocalcemia [9]. The late elimination half-life of 1,1-difluoroethane had a blood gamma of 240 seconds [10]. To date, there is no data on long-term abuse causing continued hypocalcemia after the drug is eliminated in a naive individual. There is currently no antidote for 1,1-difluoroethane toxicity; however, cytochrome P450 has been implicated in its metabolism as pretreatment with SKF-525F, disulfiram, or dimethyl sulfoxide prevented or delayed the toxicity in rats exposed to the compound, indicating a possible route for future intervention [9]. Healthcare providers are aware of common causes of hypocalcemia such as PTH or vitamin D abnormalities, and even less common causes such as hyperphosphatemia, acute pancreatitis, chronic kidney disease, and sepsis are widely known. However, certain populations are at risk for less common, but no less dangerous, causes such as 1,1-difluoroethane inhalation. Given the rise in popularity and ease of availability of this type of compound as a recreational drug, combined with the possibly fatal consequences of its abuse, providers should be cognizant of drugs of abuse as a potential cause of hypocalcemia.

## Conclusions

Aerosolized sprays are commonly used as recreational drugs. 1,1-Difluoroethane is a propellant commonly used in aerosol sprays and has been noted to cause severe hypocalcemia. We report for the first time 1,1-difluoroethane abuse in a patient with osteoporosis and refractory hypocalcemia. Given the rise in the popularity and ease of availability of this type of compound as a recreational drug, combined with the possibly fatal consequences of its abuse, providers should be cognizant of drugs of abuse as a potential cause of hypocalcemia.
